# Arbuscular Mycorrhizal Symbiosis: A Strategy for Mitigating the Impacts of Climate Change on Tropical Legume Crops

**DOI:** 10.3390/plants11212875

**Published:** 2022-10-27

**Authors:** Wan Teng Loo, Kah-Ooi Chua, Purabi Mazumdar, Acga Cheng, Normaniza Osman, Jennifer Ann Harikrishna

**Affiliations:** 1 Centre for Research in Biotechnology for Agriculture, University of Malaya, Kuala Lumpur 50603, Malaysia; 2 Institute of Biological Sciences, Faculty of Science, University of Malaya, Kuala Lumpur 50603, Malaysia

**Keywords:** abiotic stress, arbuscular mycorrhizae, biotic stress, food security, Glomeromycota, Leguminosae, stress mitigation

## Abstract

Climate change is likely to have severe impacts on food security in the topics as these regions of the world have both the highest human populations and narrower climatic niches, which reduce the diversity of suitable crops. Legume crops are of particular importance to food security, supplying dietary protein for humans both directly and in their use for feed and forage. Other than the rhizobia associated with legumes, soil microbes, in particular arbuscular mycorrhizal fungi (AMF), can mitigate the effects of biotic and abiotic stresses, offering an important complementary measure to protect crop yields. This review presents current knowledge on AMF, highlights their beneficial role, and explores the potential for application of AMF in mitigating abiotic and biotic challenges for tropical legumes. Due to the relatively little study on tropical legume species compared to their temperate growing counterparts, much further research is needed to determine how similar AMF–plant interactions are in tropical legumes, which AMF species are optimal for agricultural deployment and especially to identify anaerobic AMF species that could be used to mitigate flood stress in tropical legume crop farming. These opportunities for research also require international cooperation and support, to realize the promise of tropical legume crops to contribute to future food security.

## 1. Introduction

In a climate-stressed world, threats to food security will be exacerbated, resulting in widespread hunger and malnutrition, particularly in the tropics, which include parts of Asia, Africa, Central America, and the Caribbean [1]. The tropical latitudes are home to the most highly concentrated human populations [2] and yet tropical species have a higher sensitivity to changes in climate than temperate species, meaning that tropical crops, including tropical legumes, are most vulnerable to climate change [3]. Major crops that sustain global food security, such as soybean (*Glycine max*) and rice (*Oryza sativa*), have been reported to face enormous biotic and abiotic challenges as the globe accelerates toward a warmer, more unstable future [4]. Crop pests that have expanded their range due to climate change have had a significant impact on agriculture, with one notable example being the rapid spread of a nature pest host, Johnsongrass (*Sorghum halepense*), a highly invasive weed that affects the production of a variety of crops, including soybean and sorghum (*Sorghum bicolor*) in America [5]. Furthermore, the effects of rising temperatures are projected to become more complex and difficult to manage, with multiple hazards occurring concurrently, ranging from droughts and heatwaves to sea-level rise and floods. Ironically, agriculture has contributed significantly to the climate crisis in a vicious loop, accounting for roughly a quarter of global greenhouse gas emissions [6,7]. While atmospheric carbon dioxide levels have surpassed 420 parts per million (ppm) [8], the highest in recorded human history, plants can absorb the carbon from the atmosphere and replenish the organic carbon content of the soil under suitable environments [9].

The plant microbiota, which colonizes every accessible plant tissue, is a diverse but taxonomically structured community of microorganisms found in healthy plants. Plant-associated microbiomes provide fitness benefits to the plant host, such as growth and development, nutrient uptake, stress tolerance, and pathogen resistance [10]. In exchange for nitrogen, plants provide microorganisms carbon in the form of secretions, which protect them from oxygen and prevent their nitrogen-fixing activity [11]. Leguminosae (or Fabaceae) are a large flowering plant family that comprises a variety of agriculturally important legumes, including beans and peas, that have a natural symbiotic relationship with nitrogen-fixing root microorganisms and coexist harmoniously [12]. Although only a few legumes are widely utilized globally, with the protein-rich soybean being the most significant from an economic and nutritional standpoint, many lesser-known tropical legumes, such as winged bean (*Psophocarpus tetragonolobus*) and lentil (*Lens culinaris*), have recently been promoted as protein alternatives to soybean and meat [13]. More research should be focused on these potential species, especially given that the world’s major food-producing cereal species, notably rice, wheat (*Triticum aestivum*), and maize (*Zea mays*), are unable to efficiently employ or cultivate these microbial helpers in their roots [14].

One of the biggest threats to soil is intensive monoculture farming, which achieves high yields by using excessive pesticides that destroy biodiversity and fertilizers that starve the soil’s microbial populations while depleting it of essential nutrients and promoting the growth of pests and diseases [15]. Because farmers today use far more nitrogen than nature can provide through the costly Haber–Bosch process, a 20th-century invention that extracts nitrogen from the air to create synthetic fertilizers, researchers have been looking for untapped tools for sustainable agriculture, and research on soil microorganisms that may benefit or change farming has increased dramatically over the last decade [16]. Perhaps the solution to feeding an additional two billion people by the middle of the century lies beneath the soil, where thousands of microorganisms hold the key. The rhizosphere, for example, is home to some 30,000 species, with a community composition that varies depending on crop species and soil types [17]. While soil microorganisms may be the answer to soil degradation, which occurs when soil loses the physical, biological, or chemical qualities that support life due primarily to anthropogenic activities, the challenge is to understand the interactions between plants and soil microorganisms and identify the most beneficial strains [18].

Recent studies have demonstrated that soil microorganisms offer novel opportunities to increase crop yields sustainably, including the warding off of certain stresses such as pests and diseases, by enhancing nutrient accessibility, and by reducing the need for synthetic (or chemical) fertilizer. One prime example is the arbuscular mycorrhizal fungi (AMF), which are natural root symbionts that are commonly referred to as bio-fertilizers [19,20]. Several studies have demonstrated that inoculating legumes with AMF boosts their resistance to abiotic stresses such as drought, heat, salinity, and extreme temperatures [21,22]. AMF may aid in the upregulation of tolerance mechanisms in host plants while also preventing the downregulation of key metabolic pathways [23]. Furthermore, AMF can furnish host plants with vital inorganic nutrients, resulting in increased growth and yield under both unstressed and stressed conditions [19]. Despite their enormous potential to improve plant growth under climatic stresses, little is known about how AMF-induced modulations in tolerance mechanisms, as well as the crosstalk triggered to regulate plant performance, can help increase crop productivity [24,25], especially for the majority of previously overlooked leguminous species.

This review offers molecular insights into the symbiotic relationship that develops specifically between AMF and the tropical legumes that are emerging as possible plant-based protein substitutes. Apart from highlighting the beneficial role of AMF in enhancing leguminous crops under abiotic stresses, this review explores the potential application of AMF in fending off biotic challenges such as pests and diseases. Wherever possible examples from topical legumes have been used; however, for comprehensive coverage, examples from temperate legumes and from non-legume plants have been used where there is no information for any tropical legume species. The research gaps, challenges, and strategies for maximizing the potential of these bio-fertilizers are also discussed. There is a definite need for research on the AMF-mediated promotion of legume growth and productivity, but determining where to begin can be challenging.

## 2. Molecular Insight into the Establishment of Symbiosis between AMF and Tropical Legumes

**Figure 1:**
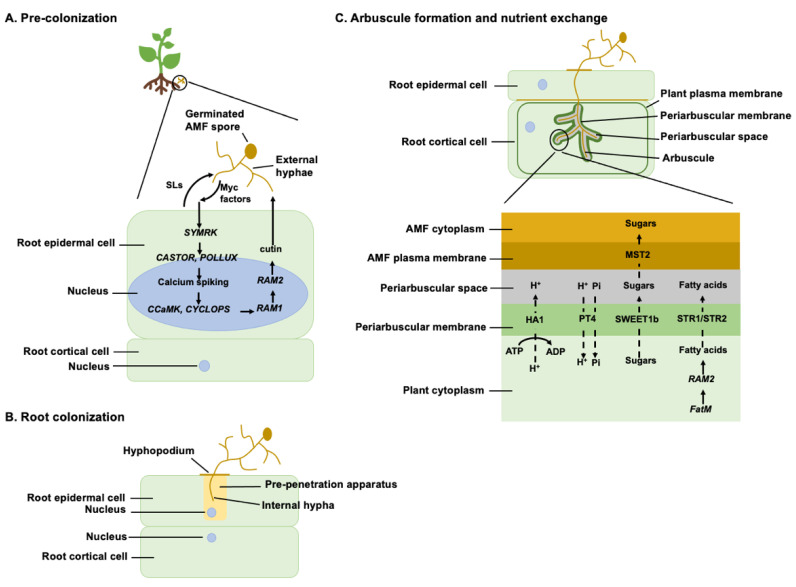
Schematic diagram showing AMF infection process. (**A**) Pre-colonization (**B**) Root colonization, (**C**) Arbuscule formation and nutrient exchange.

Most studies on legume–AMF colonization and signaling have been conducted on the model legumes, birdsfoot trefoil (*Lotus japonicus*) and barrel medic (*Medicago truncatula*), while tropical legumes such as cowpea (*Vigna unguiculata*) and pea (*Pisum sativum*) have received relatively less attention [26]. The process of establishment of symbiosis between AMF and legume plants can be categorized into three stages: pre-colonization, root colonization, and arbuscule formation and nutrient exchange (Figure 1).

### 2.1. Pre-Colonization

Chemical communication between AMF in the rhizosphere and host plant roots initiates the symbiosis process. Under phosphate deficiency, the roots of host plants secrete strigolactones (SLs), a class of phytohormones [27]. Plants produce significantly less strigolactones under high inorganic phosphate (Pi) conditions than under low Pi conditions [28]. SLs are mainly carotenoid derivatives synthesized from all-*trans*-β-carotene via a four-step enzymatic pathway [29]. The all-*trans*-β-carotene is isomerized into *cis*-β-carotene by carotene isomerase before undergoing cleavage by carotenoid cleavage dioxygenases (CCD). The *cis*-β-carotene is cleaved and modified by CCD7 (reported as *RAMOSUS5* (*RMS5*) in pea, *More Axillary Braching3* (*MAX3*) in soybean) into 9-*cis*-β-apo-10′-carotenal, which is subsequently cleaved into carlactone by CCD8 (reported as *RMS1* in pea, *More Axillary Braching4* (*MAX4*) in soybean) [30,31]. Carlactone is oxidized into SLs by members of the cytochrome P450 (CYP) family [32,33]. SLs are then released into the rhizosphere by ATP-binding cassette transporters in root hypodermal cells [34]. SLs are important for stimulating AMF hyphal branching and elongation to support root colonization between plant and AMF [27]. However, SLs are not the sole compound responsible for the establishment of root colonization. Plants with impaired SL synthesis have shown reduced AMF colonization but do not completely impede it [35].

### 2.2. Root Colonization

When AMF encounters plant-derived SLs, in response, it secretes mycorrhizal (Myc) factors. It has been proposed that these Myc factors are recognized by Lysin motif receptor-like kinases (*LysM-RLK*), which further activates *Symbiosis receptor kinase* (*SYMRK* in *L. japonica*, *Does not make infections2* (*DMI2*) in *M. truncatula*) [36]. *SYMRK/DMI2* is reported to be associated with 3-hydroxy-3-methylglutaryl-CoA reductase (HMGR), an enzyme which is involved in mevalonate production [37]. Mevalonate promotes nuclear calcium oscillations in legume root cortical cells [38]. CASTOR and POLLUX (reported in *L. japonicus and soybean*), two cation channels located on the nuclear membrane, and DMI1 (reported in *M. truncatula*), a single inner-membrane-localized channel, were also reported to play crucial roles in inducing calcium oscillations [39,40]. Calcium oscillations activate the calcium- and calmodulin-dependent protein kinase (CCaMK) [41], which phosphorylates CYCLOPS. Following that, CYCLOPS binds to a cis-element in the *Reduced arbuscular mycorrhizal1* (*RAM1 in L. japonicus, M. truncatula and soybean*) promoter and induces its transcription [41,42]. *RAM1* encodes a GRAS-domain transcription factor that regulates the expression of *RAM2*. *RAM2* encodes a glycerol-3-phosphate acyl transferase (GPAT) that is involved in the production of cutin monomers [43]. Cutin monomers are essential for hyphopodia formation in AMF [44]. Hyphopodia are specialized hyphal branches composed of lobbed cells which help in the adhesion of AMF to plant roots [43]. Mutation in *RAM1* and *RAM2* showed a reduction in AMF colonization and impaired hyphopodia formation [45]. The nucleus of the root epidermal cell in contact with the hyphopodium migrates and positions itself underneath the hyphopodium to initiate the formation of the pre-penetration apparatus, a subcellular tube-like structure [46].

### 2.3. Arbuscule Formation and Nutrient Exchange

The pre-penetration apparatus of the host plant allows AMF hypha to grow intracellularly [46]. When AMF hypha reaches the root cortical cells, it forms an arbuscule (intercellular branching hyphal structure). A plant protein, Vapyrin, plays an important role in epidermal penetration and arbuscule formation [47]. Silencing of the *Vapyrin* gene in *M. truncatula* showed impaired epidermal penetration and abolishment of arbuscule formation [48]. During the arbuscule formation, the root cortical cell undergoes a major transient reorganization and envelopes the arbuscule in a membrane known as the periarbuscular membrane (PAM) [49]. The PAM, which is continuous with the plant plasma membrane of the cortical cell, is the main interface for nutrient exchange. The area between the PAM and the AMF plasma membrane is called the periarbuscular space (PAS). HA1, a proton (H^+^)-ATPase pump, generates a proton gradient across the PAM to energize two PAM-localized proteins such as Phosphate transporter4 (PT4) and Ammonium transporter2 (AMT2) [50]. Following that, the energized PT4 imports phosphate, and AMT2 imports ammonia from the arbuscules to the plant cell. In return, plants provide carbon sources to the AMF in the form of sugars and lipids [19]. Sugars, mainly in the form of hexose, get transported to the PAS by sugar transporters such as Sugars Will Eventually be Exported Transporters1b (SWEET1b) [51]. Sugars in the PAS are transported by Monosaccharide Transporter2 (MST2) into the arbuscules [52]. AMF lack genes required to synthesize long chain fatty acids [53]. Therefore, AMF depend on host plants for important fatty acids. The involvement of plant fatty acid in arbuscule development has been demonstrated in *M. truncatula*. *Medicago truncatula* mutant lines defective in two lipid biosynthesis genes *FatM* and *RAM2* showed impaired arbuscule branching [46]. *FatM* encodes an ACP-thioesterase, which terminates fatty acid chain elongation and releases 16-carbon fatty acid. The 16-carbon fatty acid, along with CoA used as a substrate by GPAT (encoded by *RAM2*) to produce 16-carbon β-monoacylglycerol [49]. This fatty acid compound is diffused across the PAM by two half-ABC transporters, Stunted Arbuscule1 (STR1) and STR2 located at the PAM [54]. The expression of *STR1* and *STR2* in AMF is dependent on Pi concentration. High Pi represses the expression of *STR1* and *STR2*. Hence, depending on Pi supply, the AMF may starve for plant lipids.

## 3. AMF Symbiosis and Climate Change Stress Alleviation

### 3.1. Alleviation of Abiotic Stress

Numerous studies report that climate change-related abiotic stresses including heat, drought, salinity, and waterlogging impair the uptake of soil nutrients and water balance [55,56,57]. These phenomena cause crop yield to reduce significantly, resulting in increased pressure for food production. Although the use of chemical fertilizers can increase soil fertility and crop productivity, excessive use of agro-chemicals reduces the soil organic matter and quality, and the residues contribute to pollution of adjacent water bodies [58]. To circumvent these problems, farming communities are considering soil microorganism such as AMF for sustainable agricultural practice (Table 1).

#### 3.1.1. Heat Stress

Extreme heat stress affects legume growth and metabolism. Under heat stress, heat-sensitive enzymes involved in physiological processes are inhibited, causing a disruption in the cellular homeostasis; this is associated with the accumulation of harmful reactive oxygen species (ROS) [86]. This was reflected in elevated electrolyte leakage, proline, malondialdehyde (MDA), and hydrogen peroxide (H_2_O_2_) contents in heat-stressed faba bean [87] and mung bean (*Vigna radiata*) [88] plants (Table 1). Heat stress also affects the reproductive stages and yield of legumes. Heat-stressed green bean (*Phaseolus vulgaris*) plants showed delayed flowering and decreased pollen viability and pod and seed yields as compared to non-stressed plants [89]. Similarly, a 10-day exposure to 35/16 °C (day/night) during the flowering and pod development stage resulted in decreased pod yield in chickpea (*Cicer arietinum*) plants [90] (Table 1).

Studies have shown that AMF help in alleviating the negative effects of heat stress in many plants, although relevant information on AMF–legume interaction is limited. Under heat stress, the ability of plant roots to absorb water and nutrients reduces. AMF improve plant tolerance to heat mainly through enhancement of water and nutrient uptake, which in turn improves plant growth and yield under heat stress [91]. This is evident from higher water use efficiency, water holding capacity, and relative water content in AMF-inoculated maize (*Zea mays*) grown at 40 °C [92]. Meanwhile, AMF-inoculated asparagus (*Asparagus officinalis*) accumulated more of the macronutrients nitrogen (N), phosphorous (P), and potassium (K), and micronutrients such as calcium (Ca), magnesium (Mg), and iron (Fe) than non-AMF plants under heat stress [93]. In the model legume plant *M. truncatula*, a night temperature elevated by 1.53 °C negatively affected growth. Inoculation with the AMF *Rhizophagus irregularis* mitigated the effects of heat stress and enhanced *M. truncatula* growth in terms of biomass, flower and seed number, leaf sugar, shoot zinc, and root phosphorus contents [83] (Table 1). While the mechanisms have not been reported for any legume species, AMF have been associated with improved photosynthetic capacity, stomatal conductance, and transpiration rate in heat-stressed maize inoculated with a mixed AMF culture of *R. irregularis*, *Funneliformis mosseae*, and *F. geosporum* [94]. The improved photosynthesis in AMF-inoculated plants could be attributed to protective effects from the symbiotic fungi against oxidative damage caused by high temperature. Under heat stress, mycorrhizal plants often exhibit enhanced activities of various antioxidant enzymes [93,95]. AMF *Septoglomus deserticola* and *S. constrictum* ameliorated heat stress-associated oxidative damage in tomato (*Solanaceae lycopersicum*) by reducing the levels of lipid peroxidation and H_2_O_2_, while elevating the antioxidant enzyme activities in root and leaves [96].

#### 3.1.2. Drought Stress

The impacts of drought on legume growth and yield involve a series of complex processes. Legumes respond directly to drought stress by triggering stomatal closure to prevent water loss through transpiration. This is evident from studies on soybean and green bean that reported a decrease in stomatal conductance and transpiration rate during a drought stress treatment [21,62]. Stomatal closure limits the CO_2_ intake and subsequently inhibits photosynthesis. In a drought treatment, a drought-sensitive soybean cultivar Anta82 experienced reduced photosynthesis rate after 3 days [21]. As a result of reduced photosynthesis under drought conditions, legume growth and yield are strongly inhibited. Reduction in various growth and yield parameters were also reported in drought-stressed green bean [62], soybean [21,60,61], and chickpea [65] plants (Table 1).

Studies have shown that AMF symbiosis aids in physiological regulation of legumes, which enhances their tolerance to drought. AMF colonize plant roots and develop an extensive network of extraradical hyphae in the soil surrounding the root, which help to absorb water from the soil [97] and enhance water uptake under drought conditions. Higher stomatal conductance, transpiration, and photosynthesis rates occurred in AMF-associated legumes such as soybean and green bean [21,62] (Table 1). AMF treatment on legumes is also beneficial for the uptake of nutrients: When administrated with 45% to 75% water holding capacity, mycorrhizal green bean plants had increased macro- and micronutrients including N, P, K, Mg, Fe, zinc (Zn), manganese (Mn), and copper (Cu) [63]. AMF treatment also improved the green bean yield under drought stress as seeds with higher nutrient contents including N, K, Mg, Ca, vitamin B1, folic acid, crude fiber, and protein were produced compared to non-mycorrhizal plants [62].

Under long term water deficit, stomatal closure and low CO_2_ supply alter the cellular homeostasis and disrupt the electron transport and carbon-reduction cycle [98]. Low intercellular CO_2_ leads to over-reduction of electron transport components and subsequent leakage of electron to oxygen molecules which generates ROS. A high ROS accumulation causes oxidative damage to the nucleic acids, proteins, and lipids [99]. Drought stress resulted in high superoxide radical (O_2_^−^), H_2_O_2_, and MDA accumulation in black locust which was ameliorated by AMF treatment (Table 1). Mycorrhizal black locust exhibited higher activities of the antioxidant enzymes superoxide dismutase (SOD), peroxidase (POD), catalase (CAT), ascorbate peroxidase (APX), and glutathione reductase (GR) [68]. The ROS generated during drought stress also damage the photosynthetic apparatus of plants and this was evident from the reduced photosynthetic chlorophyll pigments in chickpea under water stress. This condition was ameliorated in mycorrhizal chickpea plants reflected by enhanced chlorophylls and carotenoid content [65].

Several studies have shown that interaction with AMF altered gene expression in the host legumes in response to drought stress. To combat the ROS generated during a drought treatment, mycorrhizal black locust plants exhibited higher gene expression for antioxidant enzymes such as Cu/Zn-superoxide dismutase (*Cu/Zn-SOD*), ascorbate peroxidase (*APX*), and glutathione reductase (*GR*) in the roots, stems, and leaves or at least one of the organs [68]. This shows that AMF–legume interaction provides protection against drought by inducing the expression of genes responsible for antioxidant enzyme activities. Aroca, et al. [100] reported that AMF treatment enhanced the tolerance of green bean plants to drought through regulation of various aquaporin genes that were found to be differentially expressed in the presence of AMF. A transcriptome analysis also revealed up- and downregulation of aquaporin-related genes in the leaves and roots of drought-stressed mycorrhizal green bean plants, together with altered transcription levels in pathways for osmoregulation, DNA repair, and response to oxidative stress during drought stress [64].

#### 3.1.3. Salinity Stress

Salinity impedes the growth and decreases the yield of many legume species. Salinity tests (ranging from 50 to 250 mM NaCl) resulted in lower plant height and shoot and root biomass in green bean [70], soybean [71], pigeon pea [73], cowpea [78], and chickpea [80]. Salt stress also decreased pod yield and dry weight in faba bean and green bean [69,70] (Table 1). These negative impacts are attributed to the osmotic, ionic and oxidative stresses that are induced under high salinity [101]. Studies have shown that AMF symbiosis reduces the negative impacts of salt stress in legumes. AMF-inoculated pea [77] and green bean [70] plants exhibited greater plant height, root length, and shoot and root biomass under salinity treatment, while mycorrhizal faba bean plants had higher numbers of pods per plant and higher pod dry weight [70,77]. Other than model AMF species such as *Glomus irradicans* [70], *R. irregularis* [73], and *F. mosseae* [79], the use of native AMF inoculum from saline soil also improved the growth and development of pigeon pea (*Cajanus cajan*) plants under salt stress [72,73].

High salinity levels induce osmotic stress that impairs the root system by reducing the water availability for plant metabolic processes [102]. AMF root colonization and extension of the fungal extraradical hyphae into the soil significantly enhance the water uptake in various legumes. This was reported for legume species such as cowpea, green bean, and fenugreek, where the AMF-inoculated plants exhibited significantly higher relative water content in their leaves when compared to non-inoculated plants [70,75,78] (Table 1).

Salinity also causes an ionic stress attributed to sodium (Na^+^) and chloride (Cl^−^) ion build-up in the plant cytosol, which is toxic to the plants [103]. At the same time, other mineral ions such as K, Ca, P, and Mg are lower in legume plant cells [69,70,77,78], which can result in reduced photosynthetic pigments [70,75]. Mycorrhization of legumes alleviates the deleterious effects of salinity-induced ionic stress by reducing uptake of toxic Na^+^ and Cl^−^, which is crucial in maintaining the ionic homeostasis in legume plant cells. As a result of AMF-inoculation, faba bean [69] and pea [77] plants retained higher P, K, Mg, and Ca contents and showed improved growth compared to non-mycorrhizal plants under high salinity. Mycorrhizal legumes also retained higher pigment contents compared to non-mycorrhizal legumes in a salinity condition, indicating the role of AMF in modulating plant ion contents such as Mg, an essential component of photosynthetic pigment [69,71,72].

Salinity also causes oxidative stress attributed to the build-up of ROS such as H_2_O_2_ and O_2_^−^ which damage cellular lipid, proteins, and nucleic acids [104]. In response to salt stress, legumes produce antioxidant enzymes or antioxidant molecules to scavenge the aggressive ROS [73,105] and increase levels of proline that could reduce the oxidation of lipid [106]. AMF inoculation can enhance the salt tolerance of legumes such as green bean [70], faba bean [69], and pigeon pea [73] by elevating antioxidant enzyme levels (Table 1). Compared to non-inoculated plants, these mycorrhizal legumes showed higher levels of SOD, CAT, GR, and POD. Mycorrhization of fenugreek and grass pea plants was also associated with enhanced proline content [75,76] although levels were reduced in mycorrhizal pea plants [77]. In addition, MDA, an end-product of lipid peroxidation, was reduced in various mycorrhizal legumes such as peanut [22], pigeon pea [73], and soybean [71] (Table 1).

The complexity of AMF-induced salt tolerance in legumes has been alluded to from transcriptome studies: Transcriptome analysis identified differentially expressed genes (DEGs) that were responsible for regulation of biological processes pertinent to oxidation and reduction, oxidative stress response, cell wall, and cellular component organization in the roots of mycorrhizal peanut plants that were salt-stressed, as compared to non-mycorrhizal plants. The study also reported higher expression of peroxidase and glutathione S-transferase genes in mycorrhizal peanut plants [22]. In another study, AMF-mediated salt tolerance in *Sesbania cannabina* was associated with DEGs related to oxidation-reduction processes, photosynthesis, and several transcription factor groups. Elevated expression of genes related to SOD, CAT, POD, and GR was also observed in mycorrhizal *S. cannabina* plants, indicating the AMF role in enhancing the plant ROS-scavenging capability under salinity stress [81].

A majority of legume species form symbioses with rhizobia, which are beneficial for growth and productivity. However, the number of root nodules, nodule biomass, and leghemoglobin content in various legume plants decreases under high salinity [69,72,102,107] (Table 1). This indicates reduced nitrogen fixation as leghemoglobin is responsible for supplying oxygen to nitrogen-fixing bacteria and protecting nitrogenase against oxygen damage in the nodules [108]. AMF inoculation alleviated these salt stress impacts in the legumes faba bean and soybean, which demonstrated enhanced nodulation and higher nitrogenase activity in their nodules [69,71,72]. Mycorrhization also led to higher accumulation of trehalose that can act as osmoprotectant in the nodules of salt-stressed pigeon pea [72] plants. Nonetheless, high salinization reduces AMF root colonization and spore counts in the rhizosphere, as reported in faba bean [69], grass pea [76], and pigeon pea [74] plants.

#### 3.1.4. Waterlogging Stress

Legumes are very susceptible to waterlogging stress and do not thrive under inundated conditions. Under waterlogged conditions, soybean [109], cowpea [110], faba bean, grass pea, and lupins [111] suffered significant growth reduction and yield loss. Waterlogging causes air in the soil to escape and available oxygen is greatly reduced, resulting in a hypoxic condition in the rhizosphere that inhibits plant root respiration. Although plants can maintain energy production through anaerobic respiration, the process also accumulates toxic metabolites such as lactic acid, ethanol, aldehydes, and various ROS species [112,113]. High ROS levels cause oxidative damage to the cell membrane [114] and photosynthetic apparatus [110]. This is evident from the high cell membrane injury in green bean plants waterlogged for 7 days, and the significant decrease in stomatal conductance, chlorophyll contents, transpiration, and photosynthesis rates, as well as final seed yield in waterlogged mung bean [115] and cowpea [110]. Waterlogging also prevents root uptake of essential nutrients from the soil such as nitrogen and minerals. This leads to a nutrition imbalance which also contributes to reduced growth and yield loss in legumes. Although faba bean plants survived a 20-day waterlogging treatment, the plants showed severe reduction in total nitrogen uptake, in addition to decreased seed and biomass production [116].

Studies have shown AMF symbiosis to help plants to survive waterlogging stress. However, waterlogged soil creates an anaerobic condition which is unfavorable for AMF which are obligate aerobes [117] and decreases root colonization in various plants [117,118]. Nonetheless, some AMF such as *Gigaspora* species in rice ecosystems were found better adapted to semi-aerobic and anaerobic soils [119]. These AMF were shown to provide protective effects and enhance the growth of various plants under waterlogging stress. Although there is limited study on the role of AMF in enhancing legume growth and survival in waterlogged conditions, it was shown that the AMF root colonization of green bean plants was not affected by repeated short term flooding [85]. When AMF-colonized green bean plants were treated with short term flooding, they exhibited enhanced growth in terms of root dry weight [85] (Table 1). This indicates the potential of AMF in alleviating the negative effects of waterlogging in legumes. As climate change is associated with more extremes in weather, including flooding, as well as predicted rises in sea levels in the tropical regions, it will be valuable to explore AMF that are able to mitigate waterlogging stress in tropical legumes.

### 3.2. Alleviation of Biotic Stress

The role of AMF in the alleviation of biotic stresses in tropical legumes has received much less attention compared to abiotic stresses [120]. The following are examples of pests and diseases that affect legumes and for which AMF have been reported to relieve symptoms (also summarized in Table 2). From the limited number of available reports, it can be suggested that much further research is needed in this area.

#### 3.2.1. Bacterial Pathogens of Legumes

AMF colonization triggers mycorrhizal-induced resistance (MIR) in host plants, and activates immune responses such as callose deposition, cell wall thickening, and production of ethylene, ROS, and antimicrobial compounds [137]. Studies have shown that AMF colonization reduces disease symptoms of several bacterial pathogens in tropical legumes. *Pseudomonas syringae* pv. Glycinea (*Psg*) is a pathovar that causes bacterial blight in soybean plants, producing effector proteins that suppress the host plant’s immunity and resulting in leaf chlorosis and necrosis, lesions on soybean pods, and discoloration of the stem [122]. Inoculation of soybean with AMF species *Entrophospora infrequens* reduced *Psg* colonization of plants seven-fold compared to non-AMF plants [121]. The higher uptake and transfer of N and enhanced biomass production induced by *E. infrequens* was suggested to have an important role in improving the immunity of soybean plants. Another bacterial pathogen of legumes, *Xanthomonas campestris* pv alfalfae, is a pathovar that is responsible for leaf spots in several legume plants, including alfalfa and *M. truncatula* [124]. Inoculations of AMF species *Glomus intraradices* (reclassified as *R. intraradices*), *Glomus versiforme* (reclassified as *Diversispora versiformis*), and *Gigaspora gigantea* in *M. truncatula* infected with *X. campestris* reduced disease symptoms in the leaves and upregulated defense-related genes compared to non-AMF plants [123].

#### 3.2.2. Fungal Pathogens of Legumes

Soil-borne fungal pathogens compete with AMF for infection sites and the presence of arbuscules in plant host cells can prevent invasion by hyphae of fungal pathogens [138]. Charcoal root rot is a soilborne disease caused by the broad-range fungus *Macrophomina phaseolina*. This pathogen infects the roots and lower stem of over 500 plant species including legumes such as peanut, soybean, and chickpea [139]. Multiple AMF species have shown to reduce the symptoms caused by *M. phaseolina* in legumes. In chickpea plants, inoculation with individual AMF species *Glomus fasciculatum* (reclassified as *Rhizophagus fasciculatus*), *Glomus constrictum* (reclassified as *S. constrictum*), *G. intraradices* (reclassified as *R. intraradices*), *Gigaspora margarita*, or *Acaulospora* sp. were able to reduce root-rot severity and increase plant growth, chlorophyll content, and the number of pods compared to non-AMF plants [127,128]. AMF species *R. irregularis* has been reported to upregulate pathogenesis/disease-resistant genes and increase lignin production in soybean plants under *M. phaseolina* infection compared to non-AMF plants [126]. This response reduces disease incidence and severity, leading to an improvement in plant growth and yield [125]. Various wilt diseases in legumes are caused by fungi, including Fusarium wilt in pigeon pea due to infection with *Fusarium udum*. Symptoms of infected plants include chlorosis, leaf and stem drooping, and wilting [130]. Inoculation of pigeon pea with AMF species *G. fasciculatum* (reclassified as *R. fasciculatus*) was able to significantly reduce wilting severity, and increased plant height, shoot dry weight, and phosphorous content compared to non-AMF plants [129]. Another wilt infection of pea that also leads to severe root rot and seedling damping off is caused by *Aphanomyces euteiches* [133]. This pathogen also affects a large range of other legume plants. Inoculation of pea with AMF species *G. fasciculatum* (reclassified as *R. fasciculatus*) and *G. intraradices* (reclassified as *R. intraradices*) significantly reduced spore production and root-rot severity compared to non-AMF plants [133,135,140]. Inoculation with *G. intraradices* (reclassified as *R. intraradices*) in pea also delayed and reduced the activity of *A. euteiches* enzymes such as glucose-6-phosphate dehydrogenase, phosphoglucomutase, and peptidase, which are essential for pathogenesis of *A. euteiches* [132,134]. *Phytophthora sojae* is a host-specific pathogen and only infects soybean. The disease causes damping off in seedlings, and root rot and stem lesions in mature plants [141]. Inoculation of AMF species *G. intraradices* (reclassified as *R. intraradices*) reduced oxidative damage in soybean plants infected with *P. sojae* by decreasing H_2_O_2_ content and increasing jasmonic acid content, glutathione reductase activity, and the metabolism of nitrogen and carbon compared to non-inoculated controls [131].

#### 3.2.3. Nematode Infections of Legumes

AMF colonization leads to altered composition of exudates from the roots of host plants, which may affect nematode motility and infection [142]. The effect of root exudates on nematodes is species-dependent, so the degree of protection will be variable [143]. *Heterodera cajani* is a nematode species that mainly infects pigeon pea but it is also able to infect other legumes such as cowpea and mung bean, causing root galling, stunting, and leaf chlorosis [144]. Inoculation with AMF species *F. mosseae* reduced the *H. cajani* population by over 40% in infected pigeon pea plants and increased plant length, shoot dry weight, and phosphorous content compared to non-inoculated plants [129]. Another nematode species that can infect more than 3000 plant species including legumes such as cowpea and chickpea is *Meloidogyne incognita*. Found in tropical and subtropical regions, *M. incognita* causes root galling, reduced growth and leaf chlorosis [145]. Inoculation with individual species of AMF species *G. fasciculatum* (reclassified as *R. fasciculatus*), *G. constrictum* (reclassified as *S. constrictum*), *G. intraradices* (reclassified as *R. intraradices*), *G. margarita* or *Acaulospora* sp. in chickpea infected with *M. incognita* increased plant height, fresh and dry weight, yield, and chlorophyll content together with a reduced nematode population and less root galling than non-AMF plants [127,128].

#### 3.2.4. Insect Pests of Legumes

In addition to bacterial, fungal, and nematode pathogens, inoculation of some legume plants with AMF species has been shown to reduce damage caused by insects. Well established AMF colonization increases biosynthesis of jasmonates in host plants [146]. Jasmonates regulate production and emission of volatile organic compounds such as terpenoids which repel pests or attract predators of pests [147]. Inoculation of *M. truncatula* with AMF species *R. irregularis* reduced phloem ingestion by the pea aphid *Acyrthosiphon pisum*, resulting in higher carbon content in the plants compared to non-inoculated plants [135]. Another example is inoculation with AMF species *G. intraradices* (reclassified as *R. intraradices*) in *Vigna mungo* (black gram) exposed to tobacco cutworm (*Spodoptera litura)*. AMF-inoculated plants showed higher lignin content and plant biomass compared to non-inoculated plants [136].

## 4. AMF and Tropical Legumes for Sustainable Agriculture: Challenges and Prospects

From global warming to soil degradation and biodiversity loss, the world is facing enormous environmental challenges. Fortunately, there is growing recognition and awareness that urgent action is required to reduce anthropogenic impacts on the earth at all levels—research, governmental, business, and individual [148]. When it comes to how climate change affects people equally, it can be profoundly unfair because those who have contributed the least to the problem, such as the poor, underprivileged, ethnic minorities, and indigenous peoples, suffer disproportionately from its effects and are less resilient to extreme change [149]. It is worth emphasizing that crop yields are not solely determined by climate; global demand, agricultural practises, and political shifts will all have an impact on how farms fare in the future [150]. Farmers, for example, who adopt sustainable farming practises and diversify their fields with the assistance of researchers from a variety of fields ranging from genetic engineering to climate modeling, would be able to contribute to maintaining food security with the proper government support. All parties, including the producers and policymakers, must play a role in inspiring a fundamental shift in the way intensive farming is carried out [151].

**Figure 2:**
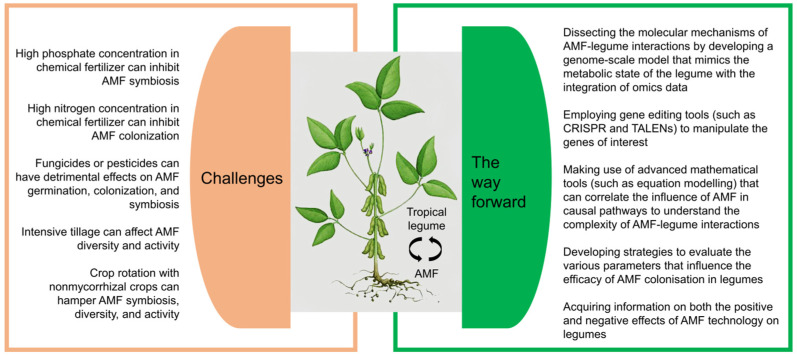
Some challenges and prospects for utilizing AMF on tropical legumes for sustainable agriculture.

Intriguingly, rising temperatures have been observed to allow some tropical crops to thrive in previously untapped growing regions further north—avocado, for example, is already grown in Sicily [152]. This may be true for some tropical legumes, but more research is needed to fully realize their potential. Numerous recent studies have shown that soil microorganisms, in particular AMF, can improve soil fertility and help plants grow more resiliently under climatic stresses [19]. Hence, researchers are now seeking to either employ effective microorganisms and/or genetically modify crops to contain their genes in order to increase crop yields, which might alter the makeup of microbial communities in the soils and enhance plant growth. For instance, AMF inoculation was found to positively alter mycorrhizal composition, resulting in increased growth of the tropical legume cowpea [153]. Nonetheless, the difficult first step is to locate elusive AMF in soil microbial communities, followed by complex biological engineering, which entails identifying the genes in AMF that stimulate efficient nitrogen fixation and then engineering them into plants in a way that the plants can use to produce the same traits [154]. Additionally, modern farming, which was introduced in the 1960s during the Green Revolution that saved billions of lives, is having a negative impact on soils and microorganisms (Figure 2), particularly through the use of high inputs such as chemical fertilizers [155,156]. The relationships between AMF and plants, particularly legumes that naturally interact with soil bacteria, require significant effort to progress (Figure 2).

AMF and other recently developed biologicals come in a variety of forms, including biological sprays that are comparable to chemical pesticides and herbicides, or seed coatings made from naturally occurring microorganisms that function as catalysts to stimulate growth and nutrient intake in crops [19,120]. Examples include the use of AMF to increase drought tolerance of several legumes, including soybean and chickpea [21,71] and the management of nematodes on soybeans using bacterial seed coatings that are damaging to the parasite [157]. These advancements are not necessarily developed to replace conventional breeding or genetic engineering, but rather to provide extra means to feed an expanding world. There is a huge opportunity to explore AMF for improving tropical legumes, especially since only approximately 10% of pesticides come from nature [158]. Soil microorganisms help crops obtain nutrients from the soil more efficiently, while also increasing disease resistance and building healthy soil structures that support livelihoods and communities. Significant reforms and efforts, however, are required at all levels, whether locally or internationally.

## 5. Conclusions

Tropical regions will be most severely impacted by climate change and the legume crops that can be grown in the tropics are of particular importance to food security as a supply of dietary protein for humans, and as a source of protein in feed and forage for fish and meat production. In conjunction with the right soil microbiota, leguminous crops are able to replenish the organic carbon content of the soil under suitable environments and this adds to their importance in a climate-changing world. It is notable that AMF, by consuming carbon-rich exudates from plant roots, are able to assist plants to maintain a source–sink balance under elevated atmospheric CO_2_. Thus, the exacerbated challenges from abiotic and biotic stresses to tropical legumes in a climate-changing world require looking to soil health and the important microbiota, not least including AMF.

Despite the large-scale production and demand for the protein-rich soybean, currently only a few other legumes are widely utilized globally. A few lesser-known tropical legumes, such as winged bean and lentil are recognized as valuable protein alternatives to soybean and meat that could be grown more widely as sustainable dietary protein sources in the future, so there is a need for more research on these potential species and their symbiotic microbes. As the community composition, comprising thousands of rhizosphere species, varies depending on crop species and soil types, there is a need to identify the most beneficial species and strains for use with the various tropical legumes, and with this, better understand the interactions between plants and soil microorganisms, for optimal mitigation of anthropogenic as well as climate change-associated soil degradation. While three stages of establishment of symbiosis between AMF and legume plants have been categorized for model legume species (Figure 1), and can reasonably be expected to be broadly the same for all legumes, it is notable that the same genes or homologs involved in these symbiotic processes may be differently named in different plant species, leading to a lack of clarity, and this is an area where the wider scientific community needs stronger coordination to facilitate further advancements in knowledge.

AMF are natural root symbionts and bio-fertilizers with the capacity to boost crop resistance to abiotic stresses such as drought, heat, salinity, and extreme temperatures. Yet the mechanisms for upregulation of tolerance responses in host plants while also preventing the downregulation of essential metabolic pathways are far from fully understood, and it is not known how much these are conserved among different plant species, most especially for the majority of previously overlooked leguminous species. Climate change will pose a host of challenges to legume crop productivity, but it is notable that several of the component stresses, namely extreme temperatures, osmotic stresses due to salinity, drought, and flooding impact many of the same fundamental metabolic pathways, and that the symbiosis between plants and AMF is able to mitigate some of the damage to plants from these multiple factors, by a few common mechanisms.

AMF mitigation of heat stress, mitigation of water stress, and mitigation of salinity stress have each been associated with enhancement of water and nutrient uptake which support photosynthetic activity and in turn plant growth and yields (Table 1). There is also an associated activation of protective metabolism to scavenge ROS, maintain osmotic balance, and reduce membrane damage, which is similarly seen with exposure to biotic stresses. However, much of this has only been demonstrated in model plant species and a very limited number of legume crop species, and there is much space for research on tropical legumes and AMF. Water stresses and salinity stress, which impact water availability, require plants to regulate transpiration via stomatal opening and closing. This will affect the movement of CO_2_ in and out of aerial parts of the plant and in turn affect photosynthesis. AMF symbiosis can mitigate water stress by increasing access to water through extraradical hyphae and it is notable that, at least in model legumes, AMF have been associated with higher stomatal conductance, transpiration, and photosynthesis rates as well as increased macro- and micronutrients including N, P, K, Mg, Fe, Zn, Mn, and Cu under water-stressed conditions. Under waterlogged conditions, however, only a few AMF species which are able to tolerate anoxic conditions are able to provide protection to their symbiotic plant partners, and there are no reported examples for legume hosts. Given the likelihood of increased flooding along with sea level rises due to climate change, this is another area where research is urgently required.

Turning to biotic stresses, AMF have been shown to reduce damage from plant pathogens and pests by various mechanisms that support plant health and immunity (Table 2), and a few studies have reported elevated gene expression of pathogenesis- and disease-resistance-related genes associated with AMF symbioses. However, as with abiotic stresses, there is a need for much further study of the underlying mechanisms and especially so for tropical legumes and their AMF.

While data and some suggestions of molecular mechanisms have been reported in model species and a few other legume species such as green bean, black locust, and chickpea, there is still much to learn concerning the mechanisms of signaling between AMF and plants that could in the future be used to breed legume crops and select AMF species that can be paired for high tolerance to abiotic and biotic stresses. Ongoing transcriptome studies to characterize genes expression show the picture to be complex, and while many of the fundamental underlying plant pathways are now quite well known, the genes involved in interaction with AMF are much less well characterized, even for model species and not at all for any tropical legume species.

AMF as a plant health promoting biofertilizer can mitigate many of the anticipated abiotic and biotic stresses associated with climate change. Their judicial application can help to reduce the amounts of chemical fertilizers required for more sustainable farming, and as the body of knowledge on the AMF of tropical legumes grows, there is much promise for their role in improving the production and availability of plant proteins in the tropics. It is noted, however, that such research requires a recognition of this need, and the will to provide support to those in less well-developed countries of the tropics, both to encourage more sustainability and self-sufficiency in food supply, and also to reduce pressures on shrinking biodiversity and environmental degradation. Supportive policies from international bodies, and not just national governments, are needed to encourage the implementation of sustainable practices, as well as to support urgently needed research. International cooperation and coordination will be needed to optimize the crops planted in the future, in which tropical legume species should strongly feature. Moreover, there is a need to recognize that the use of modern biotechnology approaches, such as gene edited crops, can be even more impactful when supported by effective microbes and this too is an area ripe for future study.

## Figures and Tables

**Table 1:** Examples of AMF alleviating abiotic stress in tropical legume crops.

Abiotic Stress	Host Plant	AMF Species	Level of Stress	Observed Abiotic Stress Effects	Observed Mycorrhizal Effects	Reference
Drought	Soybean (*Glycine max*)	*Rhizophagus clarus*	3 and 7 days	Significantly reduced growth, especially the drought-sensitive cultivar Decreased water potential, chlorophyll and carotenoid contents, photosynthetic rate, stomatal conductance, transpiration rate Higher AMF colonization in drought-stressed plants	Mycorrhizal plants showed improved growth, higher transpiration, photosynthesis, chlorophyll contents, water contents, leaf N & K contents, and stomatal conductance	[21]
	Soybean (*Glycine max*)	*Glomus mosseae* ^1^	10 days	Reduced growth Nodule experienced lower weight, nitrogenase activity and higher SOD activity Drought-stressed plants had higher lipid peroxidation, higher antioxidant enzyme activity for CAT, APX, GR but similar SOD activity	Mycorrhizal plants showed higher growth, nodule nitrogenase activity Nodule of mycorrhizal plants exhibited higher weight, nitrogenase activity Mycorrhizal plants exhibited lower lipid peroxidation, antioxidant enzyme activity except for SOD Under drought stress, mycorrhizal plants depleted more soil water content	[59]
	Soybean (*Glycine max*)	*Ambispora leptoticha*	50 and 70% field capacity from 31st to 50th day after sowing	Drought-stressed plants showed lower growth, pod yield and weight, number and weight of seeds, number and weight of nodules, chlorophyll contents	Dual inoculation with AMF *Ambispora leptoticha* and *Bradyrhizobium liaoningense* showed better performance in plant growth, pod yield and weight, number and weight of seeds, number and weight of nodules, chlorophyll content	[60]
	Soybean (*Glycine max*)	*Rhizophagus clarus*, *Gigaspora gigantea*, *Funneliformis mosseae*, *Claroideoglomus etunicatum* and *Paraglomus occulum*	40, 70 and 100% field capacity	Reduction in plant growth in terms of leaf and branch numbers, lower seed yield, weight and fatty acid contents, number of root nodules	Mycorrhizal plants with or without rhizobia showed enhanced growth, higher seed yield, weight and fatty acid contents, root nodule number, relative water content	[61]
	Green bean (*Phaseolus vulgaris*)	*Glomus mosseae* ^1^	6, 12 and 18 days	Decreased growth in terms of shoot and root dry weight, leaf area and number; decreased yield in terms of number, length and weight of pod and grain number; decreased nutrient contents Decreased chlorophyll pigment contents, photosynthetic rate, transpiration rate and stomatal conductance AMF colonization increased at 6 to 12 days drought treatment but decreased at 18 days	Mycorrhizal plants showed improved growth and yield Mycorrhizal plants exhibited higher chlorophyll content, nutrient contents (N, P, K, Ca, Mg, protein, folic acid and fiber) Both AMF and endophytic bacteria, when applied singly or together improved plant growth under water stress	[62]
	Green bean (*Phaseolus vulgaris*)	*Glomus etunicatum* ^2^, *Glomus intraradices* ^3^ and *Glomus monosporum*	Irrigation at 75, 60, and 45% of water holding capacity	Decrease in plant growth, pod yield and weight, chlorophyll content, total sugar Higher proline at low water holding capability Lower mineral and micronutrient concentrations	Mycorrhizal plants exhibited higher total sugars, chlorophyll content, total protein, N, P, K, Mg, Ca, Fe, Zn, Mn and Cu Mycorrhizal plants obtained higher plant growth, and pod yield	[63]
	Green bean (*Phaseolus vulgaris*)	*Glomus clarum* ^4^, *Acaulospora scrobiculata*, and *Gigaspora rosea*	96 h	Reduced growth, leave and root dry matters, net photosynthetic rate, stomatal conductance, transpiration rate, water use efficiency	No statistically significant difference was observed between mycorrhizal and non-mycorrhizal plants in terms of plant growth, photosynthetic rate, stomatal conductance, transpiration rate and water use efficiency AMF treatment led to differential expression and regulation of genes such as aquaporins	[64]
	Chickpea (*Cicer arietinum*)	*Claroideoglomus etunicatum*, *Rhizophagus irregularis*, and *Funneliformis mosseae*	6 weeks	Decrease in plant growth, relative water content, membrane stability, uptake of nitrogen and phosphorus, chlorophyll contents Decline in AMF colonization, mycelium, vesicles, arbuscules and spore number Decreased number of nodules, nodule weight, leghemoglobin, and nitrate reductase activity	AMF-inoculated plants showed improved growth Amendments with AMF and/or biochar showed increased relative water content and membrane stability, uptakes of nitrogen and phosphorus, chlorophyll synthesis Mycorrhizal plants showed higher number of nodules, nodule weight, leghemoglobin, and nitrate reductase activity	[65]
	Chickpea (*Cicer arietinum*)	*Rhizophagus irregularis*, *Funneliformis geosporum* and *Claroideoglomus claroideum*	Rainfed, 25, 50 and 100% water requirement and 100% water requirement only in reproductive stage	Decrease in plant growth and grain yield, seed number and weight of seeds	Co-inoculation of PGPB and AMF showed the highest performance in enhancing plant growth and grain yield, higher seed number and weight	[66]
	Cowpea (*Vigna unguiculata*)	*Rhizophagus irregularis*	Soil moisture kept at 25, 50 and 75% field capacity	Reduced growth and chlorophyll content Reduced grain yield in terms of the number, weight, and crude protein content	AMF treatment enhanced growth in terms of root, shoot and total plant dry weight, chlorophyll content under moderate water deficit but performance dropped under severe water deficit AMF treatment showed slight improvement in grain yield compared to non-stressed controls AMF and nitrogen-fixing bacteria combination showed the best performance in terms of plant growth and grain yield	[67]
	Black locust (*Robinia pseudoacacia*)	*Rhizophagus irregularis*	35 to 40% field water holding capacity	Marked reduction in growth, relative water content of leaf, stem and root, chlorophyll contents Lower mycorrhizal rate Increased levels of antioxidant enzyme activity, ROS and lipid peroxidation in both leaves and roots Increased antioxidant enzyme gene expression for Cu/Zn SOD, APX and GR	AMF treatment enhanced growth in term of dry weight, relative water content of leaf, stem and root Mycorrhizal plants exhibited higher antioxidant enzyme activity, lower ROS and MDA concentrations in both leaves and roots Mycorrhizal plants exhibited higher antioxidant enzyme gene expression for Cu/Zn SOD, APX and GR in all or at least one organ out of roots, stems and leaves	[68]
Salinity	Faba bean (*Vicia faba*)	*Funneliformis mosseae*, *Rhizophagus intraradices* and *Claroideoglomus etunicatum*	50 mM and 100 mM NaCl	Decreased growth, yield, seed weight, pigment contents, K^+^ and Ca^2+^ Increased polyamines, MDA, acid and alkaline phosphatase, antioxidant enzymes, Na^+^ content Decreased nodulation, leghemoglobin, and nodule activity AMF spore count and colonization decreased	Mycorrhizal plants showed improved growth, higher number of pod plants, pod dry weight and pigment contents Mycorrhizal plants showed higher leghemoglobin and nodule activity, K^+^ and Ca^2+^ contents, increased antioxidant enzymes, polyamines AMF-inoculated plants showed higher nodule number, nodule mass, leghemoglobin, and nodule activity Mycorrhizal plants showed lower lipid peroxidation, Na^+^ content	[69]
	Green bean (*Phaseolus vulgaris*)	*Glomus irradicans*	1000, 2000, 3000 and 4000 ppm	Reduced growth and pod yield, chlorophyll concentration, leaf relative water content Higher antioxidant enzyme activity, Na^+^, Cl^−^	AMF improved the growth, biomass of shoot, pod yield, chlorophyll, and antioxidant enzyme activity AMF-infected plants showed higher leaf relative water content Similar effects were also observed in *B. megaterium*	[70]
	Soybean (*Glycine max*)	*Funneliformis mosseae*, *Rhizophagus intraradices* and *Claroideoglomus etunicatum*	200 mM NaCl in irrigation water	Reduction in seed germination, nodulation, nodule mass, nitrogenase activity, growth hormones and chlorophyll contents reduced significantly Reduced AMF root colonization MDA, H_2_O_2_ and thiobarbituric acid reactive substances (TBARS) production increased significantly	Mycorrhizal plants showed higher nodulation, nodule mass, leghemoglobin content and nitrogenase activity, chlorophyll content and auxin synthesis Mycorrhizal plants were protected from salt-induced membrane damage and showed reduced MDA, H_2_O_2_ and thiobarbituric acid reactive substances (TBARS) production	[71]
	Pigeon pea (*Cajanus cajan*)	*Funneliformis mosseae* and *Rhizophagus irregularis*	0, 60 and 100 mM	Reduced legume growth, nitrogen, and phosphorus contents of plants Reduced AMF root colonization Reduced nodulation, nodule dry weight, leghemoglobin and nitrogenase activity; higher trehalose accumulation in nodules The salinity effects on pigeon pea were more serious in salt-sensitive than salt-tolerant genotype	AMF-inoculated plants showed higher biomass, nodulation, leghemoglobin, nitrogen, and phosphorus contents Nodules of mycorrhizal plants showed the highest trehalose content Rhizophagus irregularis performed better than Funneliformis mosseae and native inoculum from saline soil	[72]
	Pigeon pea (*Cajanus cajan*)	*Rhizophagus irregularis*	0–100 mM NaCl	Decreased plant growth, AMF root colonization Salt-stressed plants showed increased superoxide radical, hydrogen peroxide, lipid peroxidation Increased levels of antioxidant enzymes and non-enzymatic antioxidant molecules	Salt-stressed mycorrrhizal plants resulted in higher biomass and antioxidant enzymatic activities and non-enzymatic antioxidants Inoculation with Rhizophagus irregularis (alone or mixed culture) showed better results than Funneliformis mossseae and native inoculum	[73]
	Pigeon pea (*Cajanus cajan*)	*Glomus mosseae* ^1^	4, 6, and 8 dS/m	Nodule number increased at 4 to 6 dS/m but decreased at 8 ds/m Nodule size and biomass declined in all salt concentrations, sharp reduction in leghemoglobin content Increased antioxidant levels, lipid peroxidation	Mycorrhizal plants were more tolerant to salinity, showed higher nodule biomass, leghemoglobin content, nitrogenase activity and antioxidant enzyme activities Mycorrhizal plants showed reduced lipid peroxidation and membrane permeability	[74]
	Fenugreek (*Trigonella foenum-graecum*)	*Glomus monosporum* ^5^, *Glomus clarum* ^4^, *Gigaspora nigra*, and *Acaulospora laevis*	0, 75 and 150 mM NaCl	Increased salinity caused lower plant growth, leaf number and water content, chlorophyll content, AMF root colonization Higher acid and alkaline phosphatase activities, higher proline content and antioxidant enzymes in AMF-inoculated plants	AMF-inoculated plants showed enhanced growth, higher chlorophyll content, higher water content, proline, antioxidant enzyme, and phosphatase levels	[75]
	Grasspea (*Lathyrus sativus*)	*Glomus mosseae* ^1^	0, 1%, 2%, 3% and 4% (*w*/*w*) sodium sulphate	Sulphate salinity stress reduced plant growth and biomass, nodule biomass, phosphorus and nitrogen contents, AMF colonization Salinity increased proline contents	Increased plant height, AM colonization, total biomass, nodules biomass, P and N concentrations, proline concentration	[76]
	Pea (*Pisum sativum*)	*Rhizoglomus intraradices*, *Funneliformis mosseae*, *Rhizoglomus fasciculatum* and *Gigaspora* sp.	Use of soil with high salinity	Salt-stressed plants had lower plant growth, higher Na^+^, lower membrane stability index, lower yield, chlorophyll content Increased sodium ion and proline content	Mycorrhizal plants exhibited lower proline, sodium ion Mycorrhizal plants had enhanced chlorophyll synthesis, lignin deposition, higher potassium, phosphorus, and magnesium ions Multispecies-based consortium AMF performed better than single species AMF and non-inoculated in salt-stressed plants	[77]
	Cowpea (*Vigna unguiculata*)	*Funneliformis mosseae*, *Rhizophagus intraradices* and *Claroideoglomus etunicatum*	200 mM NaCl	Salt stress reduced growth and biomass, leaf size and number, chlorophyll contents, leaf water content, membrane stability Salinity reduced AMF spore count and colonization Salt stress decreased potassium, magnesium, phosphorus, calcium but increased sodium, proline, and MDA Increased antioxidant enzyme activities	AMF ameliorated the impact of salt stress on plant growth AMF-inoculated plants showed enhanced antioxidant enzyme activities and membrane stability, increased uptake of mineral elements, higher chlorophyll contents, higher leaf water content, higher proline	[78]
	Alfafa (*Medicago sativa*)	*Funneliformis mosseae*	1.4 (control), 7 and 12 dS/m soil salt concentration	Decreased plant growth and biomass, root soluble nitrogen, potassium, and calcium Salinity caused an increased sodium concentration, reduced potassium, and calcium	Mycorrhizal plants exhibited significantly higher biomass, root sugar and nitrogen content and remobilization, reduced sodium but increased potassium and calcium	[79]
	Chickpea (*Cicer arietinum*)	*Funneliformis mosseae*	0–100 mM NaCl	Reduced plant biomass, AMF colonization, calcium, and silicon contents Significant increase of ROS, MDA, indicating higher ionic leakage	AMF colonization and silicon treatment improved plant biomass and growth AMF-inoculated plants showed upregulated antioxidant enzymes and ascorbate-glutathione cycle while silicon reduced accumulation of stress metabolites more efficiently	[80]
	Sesbania Pea (*Sesbania cannabina*)	*Glomus mosseae* ^1^	200 mM NaCl	Reduced growth and biomass	Enhanced growth and biomass Enriched GO functional term for oxidation-reduction process, with DEGs associated with photosynthesis, ROS scavenging in both enzymatic and non-enzymatic pathways	[81]
	Peanut (*Arachis hypogaea*)	*Rhizophagus irregularis* and *Funneliformis mosseae*	200 mL of 200 mM NaCl at 2-day intervals	Lower plant growth in terms of root weight and length, shoot weight, chlorophyll content, relative water content of leaf Higher H_2_O_2_ and MDA were detected Increased antioxidant enzyme activity, osmolyte concentration Regulation of genes for stress response, oxidation-reduction, proline catabolism, cell wall biogenesis and so on	Enhanced growth, higher photosynthetic rate, leaf relative water content, osmolyte accumulation but lower leaf relative electrolyte conductivity Increased antioxidant enzyme activities but reduced MDA concentration Increased peanut yield, protein content in kernel AMF inoculation helped in regulation of genes responsible for oxidation-reduction process, pyruvate transport, carbohydrate metabolic process, and cell wall biogenesis and cell growth	[22]
Heat	Mung bean (*Vigna radiata*) and cashew (*Anacardium occidentale*)	*Glomus intraradices* ^3^	22, 30 and 38 °C	Low plant growth in term of shoot dry weight and root growth for both mung bean and cashew plants High temperature slowed down AMF infection in mung bean No AMF infection occurred in cashew plants at 38 °C Low AMF spore germination	Higher plant growth, enhanced root growth with AMF inoculation	[82]
	Barrel medic (*Medicago truncatula*)	*Rhizophagus irregularis*	Average increase at 1.53 °C	Reduced plant growth in terms of shoot and root biomass, flower and seed number, leaf sugar concentration, root sucrose concentration, shoot Zn and root P concentration Night warming increased AMF root colonization but not arbuscule number	Enhanced plant growth in terms of root biomass, flower number, leaf sugar concentration, shoot Zn and root P and Ca concentration Increased expression of some sucrose synthase genes, but decreased expression of the rest	[83]
	Soybean (*Glycine max*)	*Glomus versiforme* ^6^	18.2, 21.6, 25 °C	Reduced nodule weight and AMF colonization	Nodule number increased in AMF-inoculated plants	[84]
Waterlogging	Snap bean (*Phaseolus vulgaris*)	*Glomus intraradices* ^3^, *Etrophospora columbiana*, *Gigaspora margarita* and *Gigaspora rosae*	Periodic 8h flooding weekly	Reduced plant growth in terms of root dry weight Periodic flooding and subsequent draining had minimal effects on AMF root colonization	Improved growth in term of root dry weight compared to non-inoculated plants	[85]

^1^ Glomus mosseae has been reclassified as Funneliformis mosseae, ^2^ Glomus etunicatum has been reclassified as Claroideoglomus etunicatum, ^3^ Glomus intraradices has been reclassified as Rhizophagus intraradices, ^4^ Glomus clarum has been reclassified as Rhizophagus clarus, ^5^ Glomus monosporum has been reclassified as Funneliformis monosporus, ^6^ Glomus versiforme has been reclassified as Diversispora versiformis.

**Table 2:** Examples of AMF alleviating biotic stress in tropical legume crops.

Biotic Stress	Pathogen Species	Observed Biotic Stress Effects	Host Plant	AMF Species	Observed Mycorrhizal Effects	References
Bacteria	*Pseudomonas syringae* pv. glycinea	Leaf chlorosis, lesions on soybean pods and discoloration of the stem	Soybean (*Glycine max)*	*Entrophospora infrequens*, *Funneliformis mosseae*, *Claroideoglomus claroideum* and *Racocetra fulgida*	*Entrophospora infrequens* greatly reduced *Pseudomonas syringae* colonization	[121,122]
	*Xanthomonas campestris* pv. alfalfae	Water-soaked leaves which develop into dark brown spots	Barrel medic (*Medicago truncatula)*	*Glomus intraradices* ^1^*, Glomus versiforme* ^2^ and *Gigaspora gigantea*	Upregulation of defense related genes Reduced disease symptoms and bacterium population in leaves	[123,124]
Fungi	*Macrophomina phaseolina*	Caused root rot, and reduced root biomass and length Reduced aerial biomass, number of pods and leaves, plant height and greenness index	Soybean (*Glycine max)*	*Rhizophagus irregularis*	Increased pod number, plant height and root biomass Reduced disease incidence and severity	[125]
			Soybean (*Glycine max)*	*Rhizophagus irregularis*	Upregulation of pathogenesis/disease-resistance proteins Increased lignin production	[126]
			Chickpea (*Cicer arietinum)*	*Glomus fasciculatum* ^3^*, Glomus constrictum* ^4^*, Glomus intraradices* ^1^*, Gigaspora margarita, Acaulospora* sp. and *Sclerocystis* sp.	Increased plant height, fresh and dry weight, pod number Increased chlorophyll and NPK content Reduced root-rot index	[127]
			Chickpea (*Cicer arietinum)*	*Glomus intraradices* ^1^	Increased shoot dry weight, number of pods and root nodules Increased chlorophyll and NPK content Reduced root-rot index	[128]
	*Fusarium udum*	Chlorosis, leaves and stem drooping, and wilting	Pigeon pea (*Cajanus cajan*)	*Funneliformis mosseae*	Increased plant height, shoot dry weight, number of root nodules Reduced wilt index	[129,130]
	*Phytophthora sojae*	Increased H_2_O_2_ content Increased JA content, GR activity	Soybean (*Glycine max*)	*Glomus intraradices* ^1^	Decrease H_2_O_2_ content Increased JA content, GR activity, and metabolism of N and C	[131]
	*Aphanomyces euteiches*	Severe root rot, seedling damping off and wilting	Pea (*Pisum sativum)*	*Glomus intraradices* ^1^	Reduced oospore production and downward growth of pathogen in roots Delayed and shortened parasitic phase of *Aphanomyces euteiches*	[132,133]
			Pea (*Pisum sativum)*	*Glomus intraradices* ^1^	Increased shoot and root dry weight Reduced root rot severity	[133,134]
Nematode	*Heterodera cajani*	Reduced plant height, shoot dry weight and number of root nodules	Pigeon pea (*Cajanus cajan*)	*Funneliformis mosseae*	Increased plant height, shoot dry weight, number of root nodules Reduced nematode population and wilt index	[129]
	*Meloidogyne incognita*	Reduced plant height, biomass, yield and root nodules Reduced chlorophyll and NPK content Caused root rot and galling	Chickpea (*Cicer arietinum)*	*Glomus fasciculatum* ^3^*, Glomus constrictum* ^4^*, Glomus intraradices* ^1^*, Gigaspora margarita, Acaulospora* sp. and *Sclerocystis* sp.	Increased plant height, fresh and dry weight, pod number Increased chlorophyll and NPK content Reduced root galling and nematode multiplication	[127]
			Chickpea (*Cicer arietinum)*	*Glomus intraradices* ^1^	Increased shoot dry weight, number of pods and root nodules Increased chlorophyll and NPK content Reduced root galling and nematode multiplication	[128]
Insect	*Acyrthosiphon pisum*		Barrel medic (*Medicago truncatula)*	*Rhizophagus irregularis*	Reduced phloem ingestion Increased carbon content in host plants	[135]
	*Spodoptera litura*	Increased activity of ROS scavenging enzymes and defense related metabolites Reduced total plant biomass	Black gram (*Vigna mungo*)	*Glomus intraradices* ^1^	Increased activity of ROS scavenging enzymes and defense related metabolites Increased total plant biomass	[136]

^1^ Glomus intraradices has been reclassified as Rhizophagus intraradices, ^2^ Glomus versiforme has been reclassified as Diversispora versiformis, ^3^ Glomus fasciculatum has been reclassified as Rhizophagus fasciculatus, ^4^ Glomus constrictum has been reclassified as Septoglomus constrictum.

## Data Availability

Not applicable.
